# Lactate Induces Cisplatin Resistance in *S. cerevisiae* through a Rad4p-Dependent Process

**DOI:** 10.1155/2020/4971525

**Published:** 2020-10-23

**Authors:** Leslie Amaral, Filipa Mendes, Manuela Côrte-Real, Maria João Sousa, Susana Rodrigues Chaves

**Affiliations:** Centro de Biologia Molecular e Ambiental, Departamento de Biologia, Universidade do Minho, Campus de Gualtar, 4710-057 Braga, Portugal

## Abstract

Cisplatin is a widely used antineoplastic agent that has DNA as the main target, though cellular resistance hampers its therapeutic efficacy. An emerging hallmark of cancer cells is their altered metabolism, characterized by increased glycolysis even under aerobic conditions, with increased lactate production (known as the Warburg effect). Although this altered metabolism often results in increased resistance to chemotherapy, it also provides an opportunity for targeted therapeutic intervention. It has been suggested that cisplatin cytotoxicity can be affected by tumor metabolism, though with varying effects. We therefore sought to better characterize how lactate affects cisplatin sensitivity in the simplified *Saccharomyces cerevisiae* model. We show that lactate renders yeast cells resistant to cisplatin, independently of growth rate or respiration ability. We further show that histone acetylation is not affected, but histone phosphorylation is decreased in lactate-containing media. Finally, we show that Rad4p, essential for nucleotide excision repair, is required for the observed phenotype and thus likely underlies the mechanism responsible for lactate-mediated resistance to cisplatin. Overall, understanding how lactate modulates cisplatin sensitivity will aid in the development of new strategies to overcome drug resistance.

## 1. Introduction

Cisplatin is a platinum-based drug used in the treatment of various solid neoplasms, such as head and neck, lung, colorectal, and ovarian cancers [1]. This chemotherapeutic drug is administered either alone or combined with other drugs [2] and, depending on the dose, can induce irreparable DNA damage leading to cell cycle arrest or cell death, usually by apoptosis [3]. Despite its effectiveness, cellular resistance still occurs, either intrinsic or acquired [4]. Resistance is a multifactorial problem, and the most studied mechanisms range from defects in mismatch repair, aneuploidy, increased cisplatin detoxification, and failure to undergo apoptosis [5, 6]. More recently, increased attention has been given to the metabolism of tumor cells and how it affects chemoresistance. Unlike normal cells, cancer cells predominantly present a glycolytic profile even in the presence of oxygen, associated with high uptake of glucose and lactic acid production [7, 8], known as the Warburg effect or “aerobic glycolysis” [9, 10]. It is postulated that this type of metabolism can lead to a faster production of ATP and accumulation of bioprecursors to fit cellular demand [11] but may also influence the response of cancer cells to therapy [12]. Indeed, a high lactate concentration in tumors is associated with bad prognosis [13]. However, the connection between metabolism and response to therapy is not always clear-cut. Indeed, tumors are inherently heterogeneous and can include both oxidative and glycolytic cells, depending, among other factors, on vascularization and oxygen availability (reviewed in [14, 15]). A “Reverse Warburg Effect” has also been proposed, whereby cells in the tumor microenvironment are induced to provide metabolites to fuel cancer cells. For instance, lactate released by glycolytic cells can be recaptured by oxidative cancer cells to use as fuel, but also function as a signaling molecule affecting chemosensitivity (reviewed in [16, 17]) [18]. Nonetheless, results are often contradictory. In particular, it has been reported that treatment with cisplatin can lead to inhibition of glycolysis [19], and that inhibition of glucose metabolism can increase sensitivity to the drug, often through increased ROS levels [20, 21]. Cisplatin treatment also decreases mitochondrial respiration in *in vitro* cultures of HT-29, HCT116, HepG2, and MDA-MB-231, followed by a decrease in glycolysis [22]. However, while some cisplatin-resistant cell lines display higher rates of glycolysis [23–26], others have increased oxidative metabolism [27, 28]. This indicates that the relation between metabolism and cisplatin sensitivity depends on the type of tumor or cell line under study, as well as experimental conditions. Since we have previously shown that cisplatin induces an active, but mitochondrial-independent cell death process in *Saccharomyces cerevisiae* [29], we took advantage of this simpler model to clarify the role of lactate in cisplatin-induced cell death.

## 2. Materials and Methods

### 2.1. Growth Conditions and Treatments

The *Saccharomyces cerevisiae* strains used in the experimental procedures and respective genotypes are listed in Table 1. Cells were grown overnight, at 30°C and 200 rpm, in synthetic complete (SC) medium (5.0 g/L ammonium sulphate ((NH_4_)_2_SO_4_), 1.4 g/L dropout mix, 1.7 g/L yeast nitrogen base w/o amino acids and w/o ammonium sulphate ((NH_4_)_2_SO_4_), and 2% glucose (w/v)). After overnight growth, cells were collected at OD (A_640_) 0.5-0.7 and transferred to new SC medium containing 2% of the indicated carbon source (glucose or lactate). The pH of all media was adjusted to 5.0-5.5. Cisplatin (CDDP; Sigma) was added afterwards to a final concentration of 600 *μ*g/mL or 450 *μ*g/mL. Cisplatin was stored in aliquots in the dark and resuspended in DMSO immediately prior to use. However, although the phenotype in relation to control was consistent, some variability in cytotoxicity between independent experiments and different cisplatin lots was unavoidable. Methyl methanesulfonate (MMS; Fluka) was used as a positive control at a final concentration of 0.1%. *α*-mating factor was used at a final concentration of 1 mg/mL in glucose-containing media, to decrease the specific growth rate by arresting cells at the G1 cell cycle phase.

### 2.2. Viability Assays

For semiquantitative viability assays, 5 *μ*L of cell suspensions and tenfold serial dilutions (10^−1^ to 10^−4^) were spotted on YPD plates (1% (w/v) yeast extract, 2% (w/v) peptone, 2% (w/v) glucose, and 2% (w/v) agar). After 2 days of incubation at 30°C, all plates were photographed using Chemidoc XRS (BioRad) and analyzed by Quantity One (BioRad).

For quantitative viability assays, 40 *μ*L of cell suspensions were spotted on YPD plates. After 2 days of incubation at 30°C, colony-forming units (c.f.u.) were counted and normalized to the OD (A_640_) values. The percentage of cell viability was then calculated in relation to T0 (considered as 100% of cell viability). Results are expressed as mean ± standard deviation. Each experiment was performed at least 3 times. GraphPad Prism 5 was used for statistical analysis.

### 2.3. Western Blot

Total cellular extracts were separated in a 12.5% polyacrylamide gel (SDS-PAGE). Following electrophoresis, proteins were transferred onto a nitrocellulose membrane at 60 mA for 90 minutes, using a semidry system (TE77X, Hoefer, USA). Membranes were blocked for 1 h in 5% nonfat dry milk and diluted in PBS-T (PBS containing 0.05% (v/v) Tween-20), under low agitation. Subsequently, for phosphorylated H2A, H3 acetylation, and Pgk1p detection, membranes were incubated overnight at 4°C with the primary polyclonal antibodies anti-*γ*H2AX (1 : 5000, Abcam), anti-acetyl-Histone H3 (Lys9) (1 : 5000, Millipore), or anti-PGK1 (1 : 5000, Molecular Probes), respectively. The next day, membranes were washed 3 × 5 minutes with PBS-T, followed by a 1 h room temperature incubation with the secondary antibodies anti-rabbit (for *γ*H2AX and acetyl-Histone H3) or anti-mouse (for Pgk1p) from Jackson Laboratories. Chemiluminescence detection was performed using the Immobilon ECL detection system (Millipore-Merck) and a Chemi-DOC XRS system (BioRad) or X-ray ortho CP-G films in an X-ray film processor (Curix 60, Agfa Healthcare).

## 3. Results and Discussion

### 3.1. Lactate Protects *S. cerevisiae* Cells from Cisplatin-Induced Cell Death

**Figure 1:**
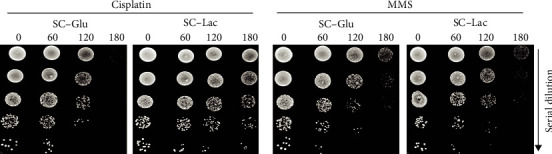
Effect of lactate on cisplatin and MMS sensitivity. Wild-type BY4741 cells were grown to early exponential phase in SC-glucose medium (pH 5.5). Cells were washed and exposed to 600 *μ*g/mL cisplatin or 0.1% MMS in SC medium containing glucose or lactate for up to 180 minutes. Cell viability was assessed by semiquantitative spot assay on YPD plates. A representative image from 3 independent experiments is shown.

To uncover the influence of lactate on cisplatin sensitivity, we assessed the viability of BY4741 cells exposed to cisplatin in medium containing glucose or lactate for up to 180 min. As a control, we also assessed viability of cells exposed to methyl methanesulfonate (MMS), a DNA alkylating agent [30]. As seen in Figure 1, lactate increased viability of cells exposed to cisplatin, but did not seemingly affect viability of cells exposed to MMS. This indicates that lactate specifically protects cells from cisplatin exposure, which may be contingent on the type of DNA damage or the pathways involved in its repair.

Indeed, MMS is a DNA-alkylating agent that causes alterations in guanine and adenine residues, resulting in base mispairing, and is predominantly repaired by the base excision repair (BER) pathway and DNA alkyltransferase [31]. In contrast, intrastrand crosslinks caused by cisplatin are mainly repaired by the nucleotide excision repair (NER) pathway [32].

**Figure 2:**
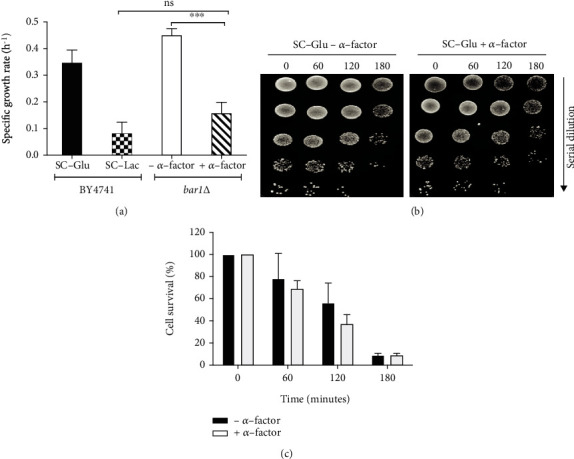
Effect of the growth rate on cisplatin sensitivity. *bar1*∆ cells were grown to early exponential phase in SC-glucose medium (pH 5.5). Cells were pretreated with *α*-mating factor for 3 hours, exposed to cisplatin for 180 minutes, and plated on YPD. (a) Calculation of the specific growth rate (h^−1^) and statistical analysis by Prism 5 (one-way ANOVA and Tukey test; ^∗∗∗^*P* < 0.0001). (b) Cell viability was estimated by semiquantitative spot assay over time. (c) Since no clear differences were observed by spot assay in (b), cell viability was quantified by c.f.u. counts in relation to time 0. Mean ± SD from 3 independent experiments is shown. Differences were not statistically significant.

Another contributing factor that may influence resistance to cisplatin could be the slower growth rate of yeast cells. Indeed, cells grow slower in lactate-containing media, which could result in additional time to repair DNA damage induced by cisplatin and underlie the observed increased resistance. To address this hypothesis, we assessed whether decreasing the growth rate in glucose-containing media affected cisplatin resistance. For this purpose, BY4741 *bar1*Δ cells were grown overnight in glucose-containing medium, pretreated with *α*-factor and exposed to cisplatin for 180 min (the *BAR1* deletion was used to decrease *α*-factor degradation). As seen in Figure 2, *α*-factor decreased the specific growth rate over the 3 h of cisplatin exposure to a level comparable or even lower to that of cells grown in lactate-containing media, consistent with a cell cycle arrest during this period. However, similar viability was observed in cells exposed to cisplatin in the presence or absence of *α*-factor (with a tendency for lower viability in the presence of *α*-factor that was not significant). This indicates that it is not the decreased growth rate but the presence of lactate that increases cisplatin resistance.

Apart from the decreased growth rate, cells grown in lactate media also display a different metabolic status, i.e., cells switch to a respiratory metabolism. As referred above, several studies report a connection between cisplatin and oxidative metabolism, though the mechanism and effects are complex. On one hand, studies show that exposure to cisplatin can itself lead to increased oxidative phosphorylation, suggesting that inhibition of tumor glycolytic metabolism could underlie decreased proliferation in response to the drug [19, 33]. On the other hand, although cisplatin-resistant and cisplatin-sensitive cells tend to display different energy metabolism, opposing effects are often found (reviewed in [12]). For instance, cisplatin-resistant ovarian and cervical cancer cells had higher rates of glycolysis whereas cisplatin-resistant lung cancer cells displayed increased oxidative phosphorylation in comparison with their cisplatin-sensitive counterparts [24–28]. To assess whether respiration affected cisplatin resistance in yeast cells, we determined whether the protective effect of lactate was still observed in the respiratory-deficient *rho^0^* strain, which lacks mitochondrial DNA.

**Figure 3:**
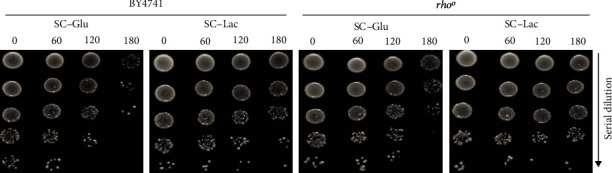
Effect of lactate on cisplatin-induced cell death of respiratory-deficient cells. Wild-type BY4741 and respiratory-deficient *rho^0^* cells were grown to early exponential phase in SC-glucose medium (pH 5.5). Cells were washed and exposed to 600 *μ*g/mL cisplatin in SC medium containing glucose or lactate for up to 180 minutes. Cell viability was assessed by semiquantitative spot assay on YPD plates. A representative image from 3 independent experiments is shown.

As seen in Figure 3, *rho^0^* cells were still more resistant to cisplatin in lactate-containing media, as observed in wild-type BY4741 cells. This indicates that it is not respiration *per se* that increases cisplatin resistance when shifting from fermentative to nonfermentative carbon sources during cisplatin exposure. These results therefore suggest that it is the lactate signaling role, in the modulation of processes other from respiration, that protects cells from cisplatin-induced death. Indeed, lactate is increasingly regarded as not just the end result of the Warburg effect but as an important molecule in driving carcinogenesis [7–10] (reviewed in [34]). While some of its effects are specific to multicellular organisms (such as increased angiogenesis), others occur at the cellular level, such as the effects of lactate on chromatin and transcription.

### 3.2. Cisplatin-Induced DNA Damage Response Is Decreased in Lactate-Containing Medium

**Figure 4:**
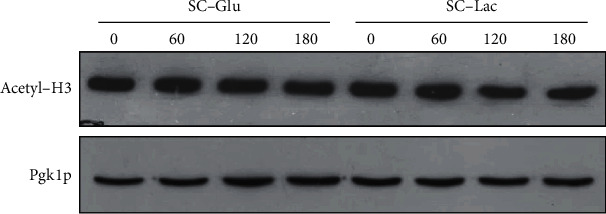
Effect of lactate on H3 acetylation during cisplatin treatment. Wild-type BY4741 cells were grown to early exponential phase in SC-glucose medium (pH 5.5). Cells were washed and exposed to 600 *μ*g/mL cisplatin in SC medium containing glucose or lactate for up to 180 minutes. H3 acetylation levels were assessed by Western blot. Pgk1p levels were used as a loading control. A representative image from 3 independent experiments is shown.

It has previously been described that lactate is a weak histone deacetylase (HDAC) inhibitor in HCT116 cells [35], resulting in increased levels of histone acetylation, associated with more relaxed chromatin. Histone acetylation has also been implicated in the regulation of the DNA damage response (DDR) in mammalian cells, and lactate has been shown to increase chromatin accessibility and DNA repair in cervical cancer cells [36]. We therefore sought to determine whether lactate could alter the levels of histone acetylation in yeast cells. For this purpose, we assessed the levels of H3 acetylation in extracts of cells exposed to cisplatin in glucose or lactate-containing media, by Western blot (Figure 4).

**Figure 5:**
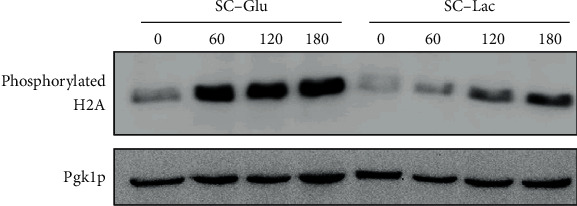
Effect of lactate on H2A phosphorylation during cisplatin treatment. Wild-type BY4741 cells were grown to early exponential phase in SC-glucose medium (pH 5.5). Cells were washed and exposed to 600 *μ*g/mL cisplatin in SC medium containing glucose or lactate for up to 180 minutes. H2A phosphorylation levels were assessed by Western blot. Pgk1p levels were used as a loading control. A representative image from 2 independent experiments is shown.

We could not detect any increase in H3 acetylation levels under our experimental conditions, suggesting that the observed cisplatin resistance in the presence of lactate is not related with increased histone acetylation. Though lactate did not seem to act by epigenetic regulation of chromatin in this case, it could still affect DDR through other mechanisms. One particularly well-known and early event that occurs after DNA damage is the phosphorylation of histone H2A or H2AX, in higher eukaryotes. In yeast, it is well established that H2A is phosphorylated in response to DNA damage, as we have previously shown in response to cisplatin [37]. We therefore assessed the levels of H2A phosphorylation in extracts of cells exposed to cisplatin in glucose or lactate-containing media, by Western blot (Figure 5).

We observed that the levels of phospho-H2A were much lower in the latter, suggestive of lower levels of damaged DNA or inhibition of the DNA damage response. However, decreased DDR should not result in increased viability; quite on the contrary, it usually results in increased sensitivity to DNA damage [38, 39]. It is, therefore, more likely that exposure to cisplatin in the presence of lactate results in decreased initial DNA damage or increased DNA repair in yeast cells.

### 3.3. Nucleotide Excision Repair Is Required for Lactate-Induced Cisplatin Resistance

**Figure 6:**
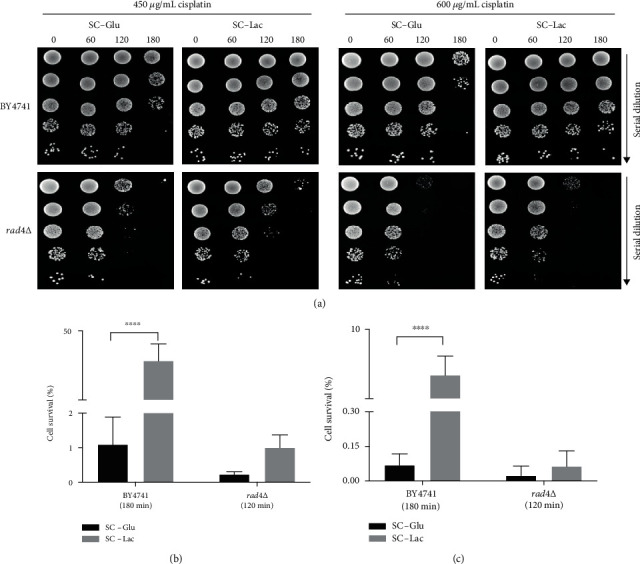
Effect of *RAD4* deletion on lactate-mediated resistance to cisplatin. Wild-type BY4741 and *rad4*Δ cells were grown to early exponential phase in SC-glucose medium (pH 5.5). Cells were washed and exposed to cisplatin in SC medium containing glucose or lactate for up to 180 minutes. (a) Cell viability was assessed by semiquantitative spot assay, (b) colony forming units (c.f.u.) of cells exposed to 450 *μ*g/mL cisplatin for 120 minutes for *rad4*Δ and 180 minutes for wild-type BY4741, and (c) c.f.u. of cells exposed to 600 *μ*g/mL cisplatin for 120 minutes for *rad4*Δ and 180 minutes for wild-type BY4741. In (b) and (c), cell viability was determined by standard dilution plate counts and expressed as a percentage of c.f.u. on YPD plates in relation to time 0. Statistical analysis by Prism 5 (two-way ANOVA and Sidak's multiple comparisons test); ^∗∗∗∗^*P* < 0.0001. Values represent mean ± SD of four independent experiments.

Taken together, the previous results indicate that the presence of lactate results in decreased initial cisplatin DNA damage or increased damage repair, which is difficult to distinguish biochemically. We therefore sought to determine whether DNA repair was required for the observed phenotype. As mentioned above, the DNA damage induced by platinum drugs is mainly repaired by the NER pathway [40], and it is well established that Rad4p is indispensable for NER activity [41]. We therefore deleted the *RAD4* gene in BY4741 cells and assessed whether lactate still rescued *rad4*∆ cells from cisplatin-induced cell death (Figure 6).

Because *rad4*∆ cells are highly sensitive to cisplatin, a lower concentration of cisplatin was also used. For both concentrations, it was possible to observe that lactate did not greatly increase the resistance of *rad4*∆ cells to cisplatin (Figure 6). Indeed, the percentage of surviving wild-type cells was significantly greater in lactate media than in glucose media after 180 min of cisplatin exposure (panels b and c). In contrast, only a minor increase was observed in the *rad4*∆ strain (measured at the 120 min time point since, after 180 min, there were virtually no viable cells exposed to either concentration of cisplatin). Taken together, though a contribution from another mechanism cannot be excluded, these results indicate that the NER pathway plays an important role in the protection from cisplatin-induced cell death imparted by lactate.

## 4. Conclusions

In this work, we show that lactate specifically increases resistance to cisplatin, independently of the cellular growth rate and respiratory capacity. This process was associated with decreased DNA damage, probably through increased NER, as Rad4p was required for the observed resistance. It is well established that repair-deficient tumor cells are particularly sensitive to cisplatin, which has been attributed to a decreased capacity to repair the DNA lesions. Our work suggests that NER deficiency may further contribute to chemotherapy efficacy by attenuating acquired resistance of tumor cells in a lactate-containing microenvironment, which warrants further investigation.

## Figures and Tables

**Table 1:** *S. cerevisiae* strains used in this work.

Strain	Genotype	Reference
BY4741	MATa *his3*Δ*1*; *leu2*Δ*0*; *met15*Δ*0*; *ura3*Δ*0*	[42]
*rho^0^*	BY4741 *rho^0^*	A. Rego
*bar1*Δ	BY4741 *bar1*Δ::*KanMX4*	Euroscarf (Germany)
*rad4*Δ	BY4741 *rad4*Δ::*URA3*	This study

## Data Availability

The data used to support the findings of this study are available from the corresponding author upon request.
